# Phase synchronization between collective rhythms of fully locked oscillator groups

**DOI:** 10.1038/srep04832

**Published:** 2014-04-29

**Authors:** Yoji Kawamura

**Affiliations:** 1 Institute for Research on Earth Evolution, Japan Agency for Marine-Earth Science and Technology, Yokohama 236-0001, Japan and Department of Mathematical Science and Advanced Technology, Japan Agency for Marine-Earth Science and Technology, Yokohama 236-0001, Japan

## Abstract

A system of coupled oscillators can exhibit a rich variety of dynamical behaviors. When we investigate the dynamical properties of the system, we first analyze individual oscillators and the microscopic interactions between them. However, the structure of a coupled oscillator system is often hierarchical, so that the collective behaviors of the system cannot be fully clarified by simply analyzing each element of the system. For example, we found that two weakly interacting groups of coupled oscillators can exhibit anti-phase collective synchronization between the groups even though all microscopic interactions are in-phase coupling. This counter-intuitive phenomenon can occur even when the number of oscillators belonging to each group is only two, that is, when the total number of oscillators is only four. In this paper, we clarify the mechanism underlying this counter-intuitive phenomenon for two weakly interacting groups of two oscillators with global sinusoidal coupling.

A system of coupled oscillators provides abundant examples of dynamical behaviors including synchronization phenomena^1^,^2^,^3^,^4^,^5^,^6^,^7^,^8^,^9^,^10^,^11^. Among them, collective synchronization emerging from coupled phase oscillators has been widely investigated not only for globally coupled systems but also for complex network systems^12^,^13^,^14^,^15^,^16^,^17^. Furthermore, the dynamical behaviors exhibited by interacting groups of globally coupled phase oscillators have been intensively investigated^18^,^19^,^20^,^21^,^22^,^23^,^24^,^25^,^26^,^27^. The appearance of the Ott-Antonsen ansatz^28^,^29^,^30^ has considerably facilitated theoretical investigations on interacting groups of noiseless nonidentical phase oscillators with global sinusoidal coupling. In addition, interacting groups of globally coupled phase oscillators as well as a system of globally coupled phase oscillators have been experimentally realized using electrochemical oscillators^31^,^32^, discrete chemical oscillators^33^,^34^, and mechanical oscillators^35^,^36^.

To study the phase synchronization between macroscopic rhythms, we recently formulated a theory for the collective phase description of macroscopic rhythms emerging from coupled phase oscillators for the following three representative cases: (A) phase coherent states in globally coupled noisy identical oscillators^37^,^38^,^39^, (B) partially phase-locked states in globally coupled noiseless nonidentical oscillators^40^, and (C) fully phase-locked states in networks of coupled noiseless nonidentical oscillators^41^. The theory enables us to describe the dynamics of a macroscopic rhythm by a single degree of freedom called the collective phase. Accordingly, different mathematical treatments were required for the physical situation in each case. The keystone of the collective phase description method for each case is the following: (A) the nonlinear Fokker-Planck equation^2^, (B) the Ott-Antonsen ansatz^28^,^29^,^30^, and (C) the Laplacian matrix^14^,^15^,^16^,^17^. Here, we note that there exist several investigations^42^,^43^,^44^,^45^,^46^,^47^,^48^ related to case (C).

In Ref. 39 for case (A) and Ref. 40 for case (B), we investigated the phase synchronization between collective rhythms of globally coupled oscillator groups. In particular, the collective phase coupling function, which determines the dynamics of the collective phase difference between the groups, was systematically analyzed for sinusoidal coupling functions. As a result, for both cases, we found counter-intuitive phenomena in which the groups can exhibit anti-phase collective synchronization in spite of microscopic in-phase external coupling and vice versa.

In this paper, using the collective phase description method developed in Ref. 41, we study the phase synchronization between collective rhythms of coupled oscillator groups for case (C). We analytically derive the collective phase coupling function for two weakly interacting groups of two oscillators with global sinusoidal coupling (see Fig. 1). We thereby demonstrate counter-intuitive phenomena similar to those found in cases (A) and (B), that is, effective anti-phase (in-phase) collective synchronization with microscopic in-phase (anti-phase) external coupling. Therefore, this paper and Refs. 39, 40 are mutually complementary and together provide a deeper understanding of the collective phase synchronization phenomena.

## Results

This section is organized as follows. First, we formulate the collective phase description of fully locked states with an emphasis on the collective phase coupling function. Second, we analyze weakly interacting groups of globally coupled two phase oscillators. Third, we perform further analytical calculations for the case of sinusoidal phase coupling. Fourth, we illustrate the collective phase coupling function for several representative cases. Fifth, we demonstrate collective phase synchronization by direct numerical simulations. Finally, we consider interacting groups of weakly coupled Stuart-Landau oscillators.

### Collective phase description of fully locked states

We consider weakly interacting groups of coupled noiseless nonidentical phase oscillators described by the following equation: 
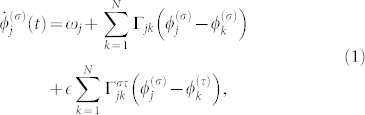
for 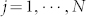 and (*σ*, *τ*) = (1, 2), (2, 1), where 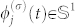 is the phase of the *j*-th oscillator at time *t* in the *σ*-th group consisting of *N* oscillators and *ω_j_* is the natural frequency of the *j*-th phase oscillator. The second term on the right-hand side represents the microscopic internal coupling within the same group, while the third term represents the microscopic external coupling between the different groups. The characteristic intensity of the external coupling is given by 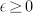. When the external coupling is absent, i.e., 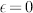, Eq. (1) is assumed to have a stable fully phase-locked collective oscillation solution^9^,^10^,^49^,^50^

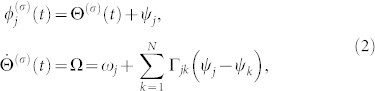
where 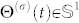 is the collective phase at time *t* for the *σ*-th group, Ω is the collective frequency, and the constants *ψ_j_* represent the relative phases of the individual oscillators for the fully phase-locked state.

When the external coupling is sufficiently weak, i.e., 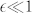, each group of oscillators obeying Eq. (1) is always in the near vicinity of the fully phase-locked solution (2). Therefore, we can approximately derive a collective phase equation in the following form^41^: 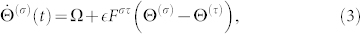
where the *collective phase coupling function* is given by 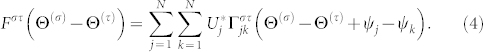
Here, 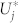 is the left zero eigenvector of the Jacobi matrix *L_jk_* at the fully phase-locked collective oscillation solution defined in Eq. (2). The Jacobi matrix *L_jk_* is given by 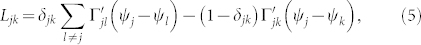
which is a Laplacian matrix^14^,^15^,^16^,^17^. That is, the Jacobi matrix *L_jk_* possesses the following property for each *j*: 
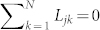
. In Eq. (5), we have used the Kronecker delta *δ_jk_* and derivative notation 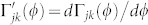. We also note that the Jacobi matrix *L_jk_* defined in Eq. (5) is generally asymmetric and weighted. Using the (*j*, *j*)-cofactor of the Jacobi matrix and the summation over the index *j*, i.e., 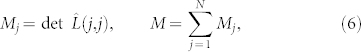
the left zero eigenvector 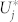 of the Jacobi matrix that takes the form of the Laplacian matrix can be generally written in the following form^41^,^42^,^43^,^44^: 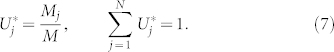
In Eq. (6), the matrix 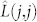 is the Jacobi matrix 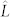 with the *j*-th row and column removed, and the cofactor *M_j_* is equal to the sum of the weights of all directed spanning trees rooted at the node *j* according to the matrix tree theorem^51^,^52^. Finally, we note that the collective phase Θ^(*σ*)^ can be written in the following form^41^: 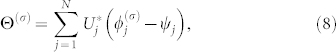
under the linear approximation of the isochron^1^,^2^,^3^,^9^,^10^,^11^.

### Interacting groups of globally coupled two phase oscillators

**Figure 1:**
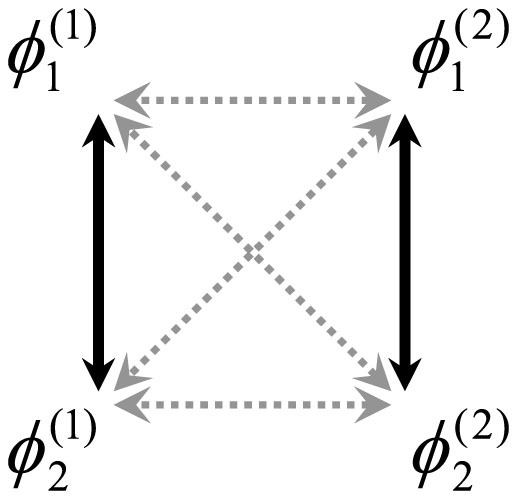
Schematic diagram of two weakly interacting groups of two oscillators with global coupling. The microscopic internal and external couplings are represented by the solid and dotted arrows, respectively, whereas the self-coupling is not shown. The phase of the *j*-th oscillator in the *σ*-th group is denoted by 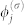.

We here analyze globally-coupled two-oscillator systems using the collective phase description method for fully locked states. We first consider weakly interacting groups of globally coupled phase oscillators. That is, the microscopic internal and external coupling functions are given by 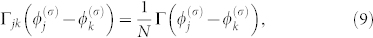


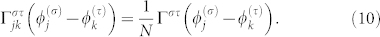
In this global coupling case, Eq. (1) is written in the following form: 
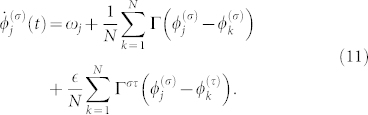
We further focus on the case in which the number of oscillators within each group is two, i.e., *N* = 2; a schematic diagram of the case is shown in Fig. 1. In this case, the internal dynamics for each group, i.e., Eq. (11) with 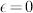, is described as follows: 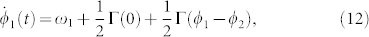


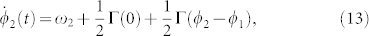
where we dropped the group index *σ* for simplicity. From Eqs. (12) and (13), we obtain the following equation by subtraction: 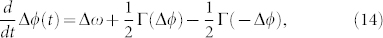
where the phase difference Δ*ϕ*(*t*) and frequency mismatch Δ*ω* are defined as 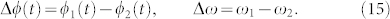
Now, we assume that Eq. (14) has a fully phase-locked collective oscillation solution. The phase difference of the stable phase-locked solution, Δ*ψ* = *ψ*_1_ − *ψ*_2_, is determined by the following equation: 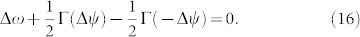
Using the phase difference Δ*ψ* obtained from Eq. (16), the collective frequency Ω is written in the following form: 
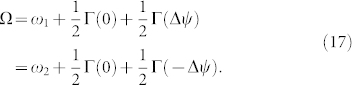
For these globally-coupled two-oscillator systems, the Jacobi matrix 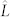 defined in Eq. (5) is given by 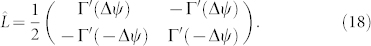
Therefore, the cofactors of the Jacobi matrix are given by 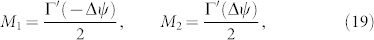
and the sum of these cofactors is written as 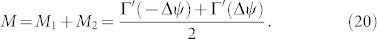
As found from Eq. (7), using these cofactors and the sum, Eq. (19) and Eq. (20), the left zero eigenvector 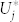 is obtained as 
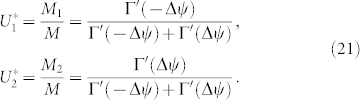
Finally, we note that the Jacobi matrix 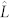 possesses not only the zero eigenvalue but also the following non-zero eigenvalue: 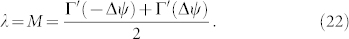
When the external coupling intensity is sufficiently small compared to the absolute value of this non-zero eigenvalue, i.e., 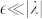, the collective phase description is valid^41^.

### Analytical formulas for the case of sinusoidal phase coupling

We here consider the case of sinusoidal phase coupling functions for both microscopic internal and external couplings. First, the microscopic internal phase coupling function is given by 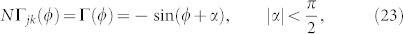
which is in-phase coupling (i.e., attractive). By substituting Eq. (23) into Eq. (16), the phase difference of the fully phase-locked state is obtained as 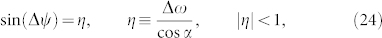
which indicates that the fully phase-locked solution emerge from a saddle-node bifurcation and exists under the condition of |Δ*ω*| < cos *α*. Owing to the in-phase coupling, i.e., Eq. (23), one solution of |Δ*ψ*| < *π*/2 is stable, and the other solution of |Δ*ψ*| > *π*/2 is unstable. Hereafter, the fully phase-locked solution indicates the stable one, |Δ*ψ*| < *π*/2. Substituting Eqs. (23) and (24) into Eq. (17), we obtain the collective frequency Ω as 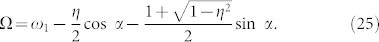
Similarly, substituting Eqs. (23) and (24) into Eq. (19), we obtain the cofactors as follows: 
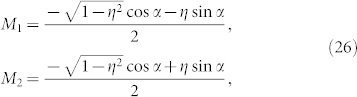
which yield 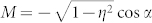. From Eqs. (21) and (26), the left zero eigenvector 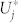 is thus written as 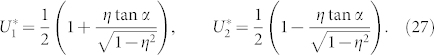
In addition, the non-zero eigenvalue *λ* defined in Eq. (22) is obtained as 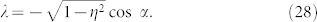
Next, the microscopic external phase coupling function is given by 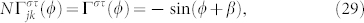
which can be either in-phase coupling (i.e., attractive) under the condition of |*β*| < *π*/2 or anti-phase coupling (i.e., repulsive) under the condition of |*β*| > *π*/2. By plugging Eqs. (24), (27), and (29) into Eq. (4), the collective phase coupling function takes the following form: 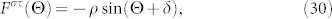
where the complex number with modulus *ρ* and argument *δ* is given by 
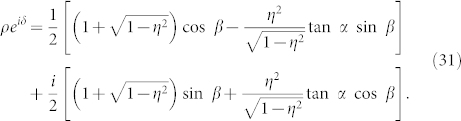
This formula is the main result of the present paper. It determines the collective phase coupling function for two weakly interacting groups of two oscillators with global sinusoidal coupling. The coupling type can be found from the real part, i.e., 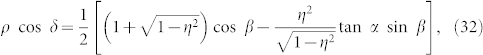
where *ρ* cos *δ* > 0 and *ρ* cos *δ* < 0 indicate in-phase and anti-phase couplings, respectively. Finally, we note that Eq. (32) possesses origin symmetry in the *α*-*β* plane.

### Type of the collective phase coupling function for representative cases

We here study the type of the collective phase coupling function for the following five representative cases.

- The first case is *η* = 0, which indicates that two oscillators within each group are identical, i.e., Δ*ω* = 0. Substituting *η* = 0 into Eq. (31), we obtain the following result: 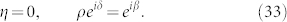
That is, the collective phase coupling function is the same as the microscopic external phase coupling function, i.e., *F^στ^* (Θ) = Γ*^στ^* (Θ) = −sin(Θ + *β*).
- The second case is 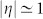, which indicates the proximity of the saddle-node bifurcation point, i.e., the onset of fully phase-locked collective oscillation. Substituting 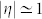 into Eq. (31), we obtain the following result: 
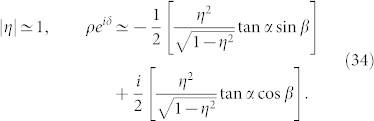
For the case of |*η*| → 1 (excluding *α* = 0), the amplitude of the collective phase coupling becomes infinity, i.e., *ρ* → ∞. Here, we note that this property for the fully phase-locked states is quite different from those for phase coherent states and partially phase-locked states^39^,^40^. For the latter two states, the amplitude of the collective phase coupling is finite at the onset of collective oscillations. This difference in the properties results from the difference of bifurcations. The fully phase-locked states emerge from saddle-node bifurcations as mentioned above, whereas the phase coherent states and partially phase-locked states emerge from supercritical Hopf bifurcations^39^,^40^.
- The third case is *α* = 0, which yields a microscopic antisymmetric internal coupling function. For this case, *η* = Δ*ω*. Substituting *α* = 0 into Eq. (31), we obtain the following result: 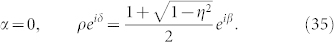
That is, the phase shift *δ* of the collective phase coupling function is the same as the phase shift *β* of the microscopic external phase coupling function.
- The fourth cases are special values of *β*. Substituting *β* = 0, ±*π*, ±*π*/2 into Eq. (31), we obtain the following results: 
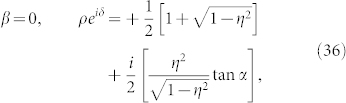

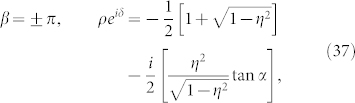

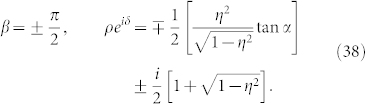
For microscopic antisymmetric external coupling functions, i.e., *β* = 0, ±*π*, the type of the collective phase coupling function coincides with that of the microscopic external coupling function. In contrast, for microscopic symmetric external coupling functions, i.e., *β* = ±*π*/2, the type of the collective phase coupling function is determined by the sign of the microscopic internal coupling parameter *α*.
- The fifth case is *β* = *α*, which indicates that the microscopic external coupling has the same phase shift as the microscopic internal one. Substituting *β* = *α* into Eq. (31), we obtain the following result: 
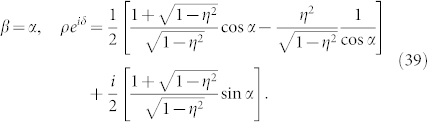
From the condition of |*α*| < *π*/2, both microscopic internal and external coupling functions are in-phase coupling. However, the type of the collective phase coupling function is anti-phase coupling under the following condition: 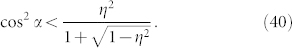
For the case of |*η*| → 1, the above condition becomes cos^2^ *α* < 1, which is satisfied for all *α* except for *α* = 0.

### Collective phase synchronization between two interacting groups

**Figure 2:**
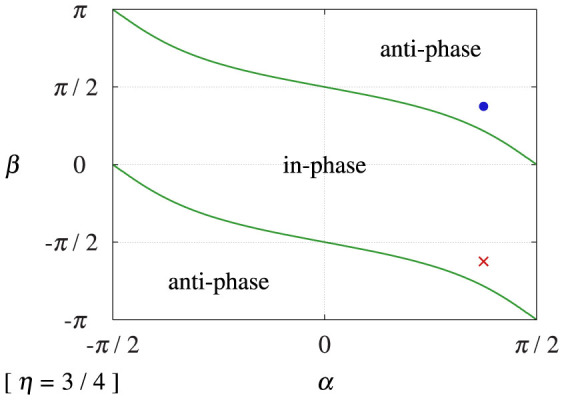
Effective type of phase coupling between collective rhythms of fully locked oscillator groups with *α* ∈ (−*π*/2, *π*/2), *β* ∈ [−*π*, *π*], and *η* = 3/4. The solid curves are analytically determined by Eq. (32), i.e., *ρ* cos *δ* = 0. The filled circle (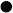) indicates *α* = *β* = 3*π*/8 corresponding to Fig. 3(a) and Fig. 4. The times sign (×) indicates *α* = 3*π*/8 and *β* = −5*π*/8 corresponding to Fig. 3(b).

**Figure 3:**
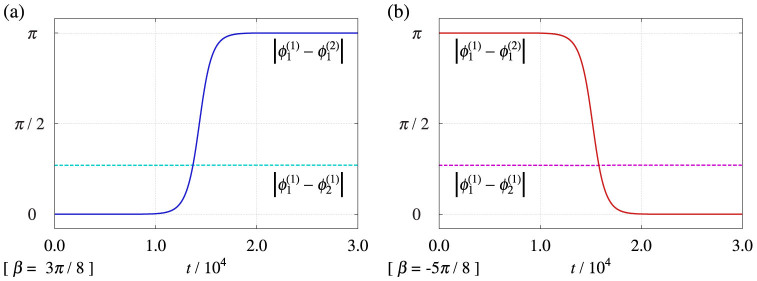
Interacting groups of phase oscillators (see Methods). Time evolution of the internal and external phase differences, i.e., 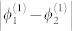 and 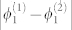. The other internal and external phase differences are approximated as 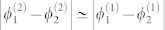 and 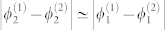, respectively. The collective phase difference is approximated as the external phase difference, i.e., 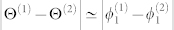. The parameters are *α* = 3*π*/8, *ω*_1_ = 3 cos(*α*)/4, *ω*_2_ = 0, and 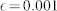. (a) Effective anti-phase collective synchronization with microscopic in-phase external coupling, *β* = 3*π*/8. (b) Effective in-phase collective synchronization with microscopic anti-phase external coupling, *β* = −5*π*/8.

Now, we study counter-intuitive cases under the condition of *η* = 3/4. The type of the collective phase coupling function is shown in Fig. 2, where the solid curves are determined by Eq. (32), i.e., *ρ* cos *δ* = 0. Here, we note that the type of the collective phase coupling function can be different from that of the microscopic external phase coupling function. Two sets of parameters, which were used in Fig. 3, are also shown in Fig. 2.

Two groups of two-oscillators exhibiting phase-locked states were separately prepared with their corresponding phases being nearly identical. Then, these states were used as the initial condition in Fig. 3(a). In spite of the microscopic in-phase external coupling, *β* = 3*π*/8, the external phase difference 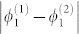 approached *π* after some time; this indicates anti-phase collective synchronization between the groups. In contrast, Fig. 3(b) shows in-phase collective synchronization between the groups in spite of the microscopic anti-phase external coupling, *β* = −5*π*/8.

### Interacting groups of weakly coupled Stuart-Landau oscillators

**Figure 4:**
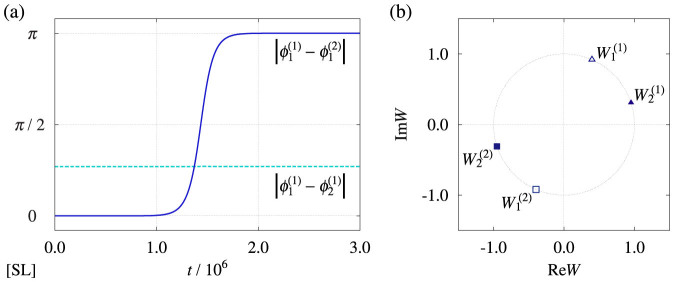
Interacting groups of weakly coupled Stuart-Landau (SL) oscillators (see Methods). Effective anti-phase collective synchronization with microscopic in-phase external coupling. The parameters are *K* = *J* = *r* cos(*a*), *c*_1_ = *c*_3_ = 0, *c*_2_ = tan(*a*), *b*_1_ = *c*_2_ + 3*r* cos(*a*)/4, *b*_2_ = *c*_2_, and 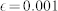, where *r* = 0.01 and *a* = 3*π*/8. (a) Time evolution of the internal and external phase differences, i.e., 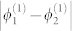 and 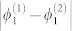. (b) Snapshot of the asymptotic state of individual oscillators, i.e., 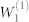, 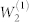, 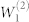, and 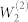.

We further consider interacting groups of globally coupled Stuart-Landau oscillators described by the following equation: 
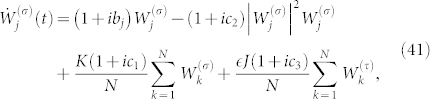
for 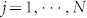 and (*ρ*, *τ*) = (1, 2), (2, 1), where 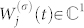 is the complex amplitude of the *j*-th limit-cycle oscillator at time *t* in the *σ*-th group consisting of *N* oscillators. The first and second terms on the right-hand side represent the intrinsic dynamics of each oscillator, the third term represents the microscopic internal coupling within the same group, and the fourth term represents the microscopic external coupling between the different groups. When the internal and external couplings are sufficiently weak compared to the absolute value of the amplitude Floquet exponent, we can approximately derive a phase equation in the following form^2^: 
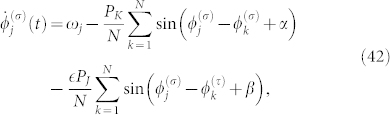
where the parameters of phase oscillators are given by 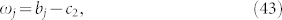


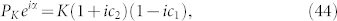


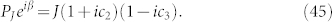
The phase of each Stuart-Landau oscillator is given by the following equation^1^,^2^,^3^,^9^,^10^,^11^: *ϕ* = arg *W* − *c*_2_ ln |*W*|. Here, we focus on the case in which the number of oscillators within each group is two, i.e., *N* = 2. Using the following constants, *r* = 0.01 and *a* = 3*π*/8, the parameters of the Stuart-Landau oscillators are fixed at *K* = *J* = *r* cos(*a*), *c*_1_ = *c*_3_ = 0, *c*_2_ = tan(*a*), *b*_1_ = *c*_2_ + 3*r* cos(*a*)/4, and *b*_2_ = *c*_2_. Under these conditions, the parameters of the p hase oscillators are obtained as *P_K_* = *P_J_* = *r* = 0.01, *α* = *β* = *a* = 3*π*/8, *ω*_1_ = 3*r* cos(*a*)/4, and *ω*_2_ = 0, which correspond to the parameters in Fig. 3(a). In particular, we note that *η* = (Δ*ω*)/(*P_K_* cos *α*) = (3*r* cos(*a*)/4)/(*r* cos(*a*)) = 3/4. The external coupling intensity is fixed at 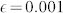. The direct numerical simulation result of Eq. (41) is shown in Fig. 4. Similarly to Fig. 3(a), Fig. 4 shows anti-phase collective synchronization between the groups in spite of the microscopic in-phase external coupling.

## Discussion

In this paper, we considered the phase synchronization between collective rhythms of fully locked oscillator groups, clarified the relation between the collective phase coupling and microscopic external phase coupling functions, analytically determined the type of the collective phase coupling function for weakly interacting groups of two oscillators with global sinusoidal coupling, and demonstrated that the groups can exhibit anti-phase (in-phase) collective synchronization in spite of microscopic in-phase (anti-phase) external coupling. The theoretical predictions were successfully confirmed by direct numerical simulations of the phase oscillator model and Stuart-Landau oscillator model.

In Refs. 39, 40, we investigated the phase synchronization between collective rhythms of globally coupled oscillator groups under two typical situations: phase coherent states in the noisy identical case^39^ and partially phase-locked states in the noiseless nonidentical case^40^. In particular, we found the counter-intuitive phenomena similar to the results in this paper. That is, weakly interacting groups can exhibit anti-phase collective synchronization in spite of microscopic in-phase external coupling and vice versa. Here, we note that these three papers considered different physical situations and utilized different mathematical methods, but arrived at the similar counter-intuitive phenomena.

We also remark that fully phase-locked states emerge from a finite number of oscillators^9^,^10^; even two is possible as actually studied in this paper. In contrast, phase coherent states and partially phase-locked states emerge from a large population of oscillators^2^; the number of oscillators is infinite in theory. From this point of view, fully phase-locked states can be more easily realized in experiments such as electrochemical oscillators^31^,^32^, discrete chemical oscillators^33^,^34^, and mechanical oscillators^35^,^36^. We hope that the counter-intuitive phenomena studied in this paper, i.e., effective anti-phase (in-phase) collective synchronization with microscopic in-phase (anti-phase) external coupling, will be experimentally confirmed in the near future and that the formula (31) will help in such experiments.

Finally, we emphasize that collective synchronization between interacting groups of coupled oscillators cannot be fully clarified by simply analyzing microscopic interactions between individual oscillators. In particular, microscopic in-phase (anti-phase) external coupling does not necessarily lead to in-phase (anti-phase) collective synchronization. As clarified in this paper, counter-intuitive phenomena can occur even when the number of oscillators belonging to each group is only two. We hope that the analytical results for the simple cases studied in this paper will provide an insight into more complex cases.

## Methods

### Numerical method for Fig. 3

We applied an explicit Euler scheme with a time step Δ*t* = 0.01 for Eq. (11) with Eqs. (23) and (29). The parameters are *N* = 2, *α* = 3*π*/8, *ω*_1_ = 3 cos(*α*)/4, *ω*_2_ = 0, and 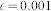 with (a) *β* = 3*π*/8 or (b) *β* = −5*π*/8. The initial values are 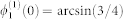 and 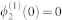 with (a) 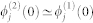 or (b) 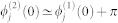.

Here, we note the accuracy and stability of the numerical method. On the right-hand side of Eq. (11), the first and second terms represent the internal dynamics of *O*(1) while the third term represents the external coupling of 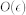. When the external coupling is sufficiently weak, the smallest time scale in Eq. (11) is *O*(1). Therefore, the explicit Euler scheme with the time step *Δt* = 0.01, which we used for the sake of simplicity and efficiency, is sufficiently accurate and stable under the parameter condition.

### Numerical method for Fig. 4

We applied an explicit Euler scheme with a time step Δ*t* = 0.01 for Eq. (41). The phase of the *j*-th oscillator at time *t* in the *σ*-th group was obtained by 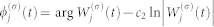. The parameters are *N* = 2, *K* = *J* = *r* cos(*a*), *c*_1_ = *c*_3_ = 0, *c*_2_ = tan(*a*), *b*_1_ = *c*_2_ + 3*r* cos(*a*)/4, *b*_2_ = *c*_2_, and 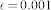, where *r* = 0.01 and *a* = 3*π*/8. The initial condition is given by 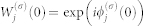, where 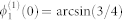, 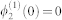, and 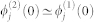.

We also note the accuracy and stability of the numerical method. On the right-hand side of Eq. (41), the first and second terms represent the oscillator dynamics of *O*(1), the third term represents the internal coupling of *O*(*K*), and the fourth term represents the external coupling of 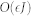. When the internal and external couplings are sufficiently weak, the smallest time scale in Eq. (41) is *O*(1). Therefore, the explicit Euler scheme with the time step *Δt* = 0.01, which we used for the sake of simplicity and efficiency, is sufficiently accurate and stable under the parameter condition.

## Author Contributions

The author designed the study, developed the theory, performed the simulation, and wrote the manuscript.
